# Interventions connecting young people living in Africa to healthcare; a systematic review using the RE-AIM framework

**DOI:** 10.3389/frhs.2024.1140699

**Published:** 2024-01-31

**Authors:** Titilola Abike Gbaja-Biamila, Chisom Obiezu-Umeh, Ucheoma Nwaozuru, David Oladele, Alexis Engelhart, Thembekile Shato, Stacey Mason, Victoria Carter, Juliet Iwelunmor-Ezepue

**Affiliations:** ^1^ Clinical Sciences Department, Nigerian Institute of Medical research, Lagos, Nigeria; ^2^ College for Public Health and Social Justice, Saint Louis University, St. Louis, MO, United States; ^3^ Department of Implementation Science, Division of Public Health Sciences, Wake Forest University School of Medicine, Winston-Salem, NC, United States; ^4^ Implementation Science Center for Cancer Control and Prevention Research Center, Brown School, Washington University in St. Louis, St. Louis, MO, United States; ^5^ School of Social Work, Saint Louis University, St. Louis, MO, United States

**Keywords:** interventions, Africa, young people, healthcare, systematic review, RE-AIM

## Abstract

**Introduction:**

Africa's young people are among the least focused groups in healthcare linkage. The disproportionally high burden of youth-related health problems is a burden, especially in developing regions like Africa, which have a high population of young people. More information is needed about factors that impact linkages in healthcare and the sustainability of health interventions among young people in Africa.

**Methods:**

A systematic literature search was performed from October 2020 to May 2022 in PubMed, CINAHL, Scopus, Global Health, and the Web of Science. Studies included in the review were conducted among young people aged 10–24 living in Africa, written in English, and published between 2011 and 2021. Results were reported according to the Preferred Reporting Items for Systematic Reviews and Meta-Analyses. Data was analyzed using narrative synthesis, synthesizing the details of the RE-AIM reporting component. Interventions were systematically compared using the Cochrane Collaboration risk-of-bias tool to evaluate the rigor of each intervention.

**Results:**

A total of 2,383 potentially relevant citations were obtained after an initial database search. Retained in the final group were seventeen articles from electronic data searches; among these articles, 16 interventions were identified. Out of the seventeen studies, nine (53%) were randomized controlled trials, three (18%) were quasi-experimental designs, and five (29%) were observational studies. At the same time, the included interventions were reported on 20 (76.92%) of the 26 components of the RE-AIM dimensions. In eastern Africa, twelve (80%) interventions were conducted, and all the interventions addressed linkage to care for young people in preventing and treating HIV. The least reported RE-AIM dimensions were implementing and maintaining interventions connecting young people to care.

**Discussion:**

Timely care remains critical to treating and preventing ailments. This review indicates that interventions created to link young people to care, especially HIV care, can help link them to health care and strengthen the programs. It is also clear that further research with more extended follow-up periods is needed to examine connections to care in all other aspects of health and to bridge the gap between research and practice in the care of young people in Africa.

**Systematic Review Registration:**

PROSPERO [CRD42022288227].

## Introduction

Linkage is vital to care as it is the earliest step in successful treatment (1). Various populations have been studied to improve the connections to healthcare in developed countries. However, in many low- and middle-income countries (LMIC) like those in Africa, studies focusing on improving links to care are lacking, especially around populations that bear the most significant burden on health (2). Africa's young people are among the least focused groups in healthcare linkage (3). According to the World Health Organization (WHO), young people are between 10 and 24 (1). Young people in Africa have limited access to developmentally based services and systems, which is rare or nonexistent today (4–6). In Africa, young people have experienced delayed treatment for some infections like HIV or Tuberculosis (TB) (6).

Africa is the youngest continent globally, with 60% of its population under 25 years (7). Evidence shows that this population will be 42% of the world's young people by 2030, which may double by 2055 (8). Today's young people in Africa are the most significant number in history, and they move towards adulthood in a very different world from generations past, paved with many challenges (9). The transition to productive and healthy adults depends on many factors, especially for young African people. Evidence has shown that young people globally are not as healthy as they seem (10, 11). Thus, the health issues of young people in Africa must be addressed now more than ever (12), as there is a disproportionally high burden of youth-related health problems in young people in Africa (12, 15), particularly in developing countries in regions like Africa, where young people hold the highest percentage in the population. Therefore, young people with prevailing health problems must be linked to healthcare. Despite the importance of this, limited studies address healthcare linkages among young people globally (13).

Linking to care is vital to improving access to clinical services, screening, counseling, and treatment, especially since access and screening are usually before diagnosis. Improved health linkage from community-level activities to medical treatment reduces and prevents disease in communities (5). The definition of “links or connections to care” differs in health scenarios, health institutions, health workers, and diseases. Some studies have defined it as the time between diagnosis and the first clinic attendance date (14). It is also defined as a completed visit to a medical provider within one month (30 days) of diagnosis (15). Studies have suggested that connecting to care varied from a few days when a patient is diagnosed with a health ailment and then linked to healthcare within a month (14–16). In this study, healthcare is defined as health improvement via the prevention, diagnosis, treatment, amelioration, or cure of disease by trained and licensed professionals (17–19). The timeline for connecting to healthcare also varied in studies, with 30 days as the highest frequency in these studies (16).

Linking and access to healthcare are different concepts within healthcare delivery and utilization (20–22), often used interchangeably. Access to care refers to the availability, affordability, and acceptability of care services, as well as the ability of individuals to reach healthcare facilities physically. Access to care is essential to healthcare systems and ensures that individuals receive the necessary medical attention to maintain and improve their health (20, 21). It refers to a person's capacity to get timely healthcare services to achieve the best possible health outcomes (20, 21). Healthcare access comprises several components, which include geographical accessibility, financial affordability, availability of healthcare facilities and services, and the absence of barriers that relate to cultural or linguistic influences (20).

Barriers to linking to care in Africa are multilayered and encompass a range of factors that hinder individuals from accessing and utilizing healthcare services. These barriers include fear of judgmental attitudes of healthcare workers (23), lack of time to access a clinic (24), lack of standardization and consistency in defining “linkage to care” (25), stigma associated with Health facilities (26, 27), service efficiency, poor provider-patient interactions, and lack of patient incentives (28), lack of accessible transportation (28), limited health insurance coverage (29), and socioeconomic factors such as poverty and lack of access to healthcare (30). Barriers also include the disparities between rural and urban healthcare, insufficient infrastructure, and the absence of programs for rural practice exposure, contributing to the obstacles (31). Lack of awareness about available services, stigma associated with seeking healthcare, or challenges navigating the healthcare system (26, 27, 32). The barriers to healthcare linkage in Africa are complex and interrelated, involving social, economic, and systemic factors. To overcome these barriers, a thorough strategy is needed to improve healthcare infrastructure, address socioeconomic disparities, standardize “linkage to care,” and implement policies to reduce stigma and enhance provider-patient interactions.

However, strategies have been used to link young people to healthcare in Africa. These include peer education, increasing youth-friendly health services training, reducing the cost of health services, addressing social risk factors, changing social norms, and promoting health by engaging the target population (33). There is a considerable gap between what is known to work and how to effectively transform these interventions into practice. Implementation science can give more clarity and scientific inquiry into what, why, and how interventions work in “real world” settings and test methods to improve them (34). In implementation science, it is generally putative to use theory or models to improve outcomes, understanding, and generalization within implementation science and many other research areas (35–38). This study seeks to identify strategies to screen, diagnose, and link young people to healthcare. Therefore, this study examines linkage strategies implemented in Africa among young people using the RE-AIM Framework.

## Methods

The protocol for this systematic review was registered on Prospero (Unique ID number: CRD42022288227). This study used an implementation model, like RE-AIM, to evaluate the approaches used in linking or connecting to healthcare (35, 37, 39). RE-AIM is intended as a conceptual model for planning, implementation, evaluation, review, and reporting of implementation science and dissemination research (37). This model is considered the “gold standard” for decision-making and guidelines (40), and this framework has been used extensively for evaluation and planning programs (35, 39, 41). Various studies have applied the RE-AIM model to plan, evaluate, and review a variety of health promotion and disease management interventions (37, 39, 41, 42). The RE-AIM framework highlights the significance of focusing on all the features of Reach, Efficacy/Effectiveness, Adoption, Implementation, and Maintenance (37).

RE-AIM theorizes that the impact of public health evidence-based intervention can succeed if there are effective interventions, the reach is extensive, there is a representative population segment by adoption of organizations, the staff is willing to ensure implementation of interventions as proposed, and the intervention is maintained over some time (43). Each of the five components is crucial to success. This is measured by public health impact and data in all five aspects, which are vital to understanding the achievements or failure of any implementation initiative, i.e., it answers the “ultimate use” question to generalize from this knowledge to other settings (42, 43). It applies to studies in LMIC, where its use is still relatively uncommon (43). This study will use the RE-AIM model to evaluate studies related to healthcare in Africa, where linking to healthcare is one of the outcomes in studies performed with young people (10–24 years).

Using the RE-AIM framework, a multi-step method was used to find, assess, and analyze the data to find existing links to care interventions that target young people 10–24 years in Africa. For this review, connecting or linking to care is defined as when a patient enters specialist care or healthcare after diagnosis or identification of a health challenge; more specifically, it is the time between the diagnosis or diagnosis date and the first clinic attendance date (14, 44, 45). Linking to healthcare is done to link or connect young people to care after being diagnosed with a health condition.

### Search strategy

**Figure 1:**
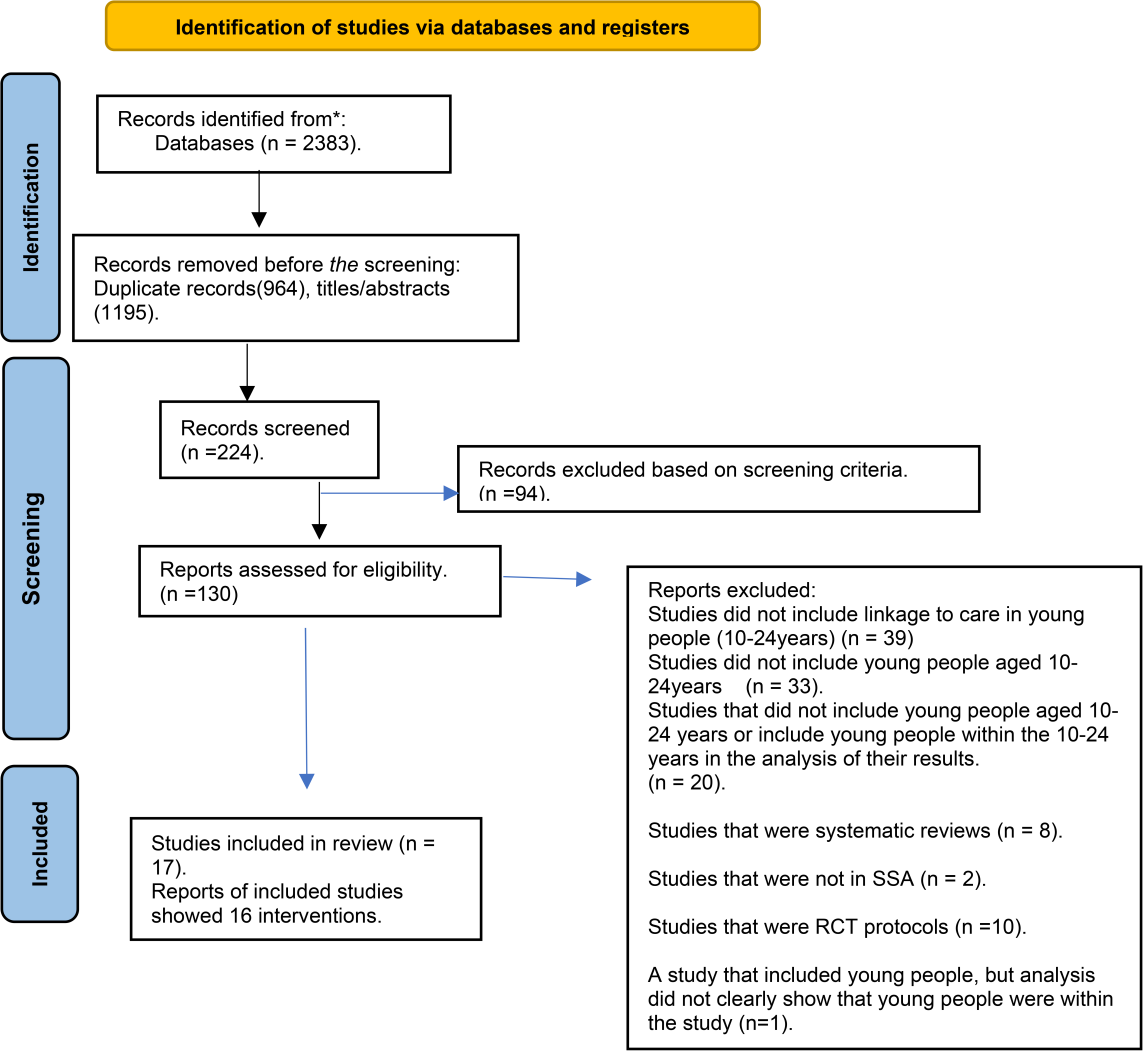
Flow diagram of the search strategy. A total of 16 unique interventions reported in 17 articles were included in the review.

A systematic literature search was executed from October 2020 to May 2022 to uncover studies published in academic journals. The search strategy was reported according to Preferred Reporting Items for Systematic Reviews and Meta-Analyses (PRISMA) guidelines (see Figure 1). Three reviewers (TG, UN, and CO) independently searched the database. The search terms were the same for each database. Check Supplementary File S1 (Table 1) for the entire search strategy. Three reviewers (TG, UN, CO) independently examined the titles and abstracts of possibly relevant papers for eligibility. The full texts of documents that matched the eligibility criteria were retrieved and separately reviewed for inclusion in the review by the two reviewers. Divergences in the screening procedure and study eligibility were discussed and resolved based on the two reviewers’ agreement.

**Table 1:** Reporting quality of included interventions (16 interventions reported in 18 papers included in the review).

	Selection bias (random sequence generation)	Selection bias (allocation concealment)	Performance bias	Detection bias	Attrition bias (incomplete outcome data)	Reporting bias (selective reporting)	Other sources of bias	% risk of bias
Ahmed Saeed et al. (2017), Malawi	High risk	Low risk	Low risk	Low risk	Unclear	Low risk	Low risk	28.6
Caroline E. Boeke et al. (2018) Uganda, Caroline E. Boeke et al. (2018b) Uganda	High risk	Unclear	Low risk	High risk	High risk	Low risk	Low risk	57.1
Shraddha Bajaria et al. (2021), Kenya	Low risk	Low risk	Low risk	High risk	High risk	Low risk	High risk	42.9
James Ayieko et al. (2019), Kenya and Uganda	Low risk	High risk	Low risk	High risk	High risk	Low risk	High risk	42.9
Lillian B. Brown et al. (2020), Kenya and Uganda	High risk	High risk	Low risk	Low risk	High risk	Low risk	Low risk	42.9
Larry W. Chang et al. (2020), Uganda	Low risk	Low risk	High risk	High risk	Low risk	Low risk	Low risk	28.6
Augustine T. Choko et al. (2015), Malawi	High risk	Unclear	Low risk	Low risk	High risk	High risk	Low risk	57.1
Nolwenn Conan et al. (2020), Malawi	High risk	High risk	Low risk	Low risk	Low risk	Low risk	High risk	42.9
Cari Courtenay-Quirk et al. (2018), Tanzania	High risk	High risk	Low risk	Low risk	High risk	High risk	Low risk	57.1
Batya Elul et al. (2017), Mozambique	Low risk	Low risk	Low risk	High risk	High risk	High risk	High risk	57.1
Hewett et al. (2016), Zambia	Low risk	Low risk	Low risk	Unclear	Low risk	Low risk	Low risk	14.3
Niklaus Daniel Labhardt et al. (2014), South Africa Lesotho	Low risk	Low risk	Low risk	Low risk	Low risk	Low risk	Low risk	00.0
Joseph.K.B. Matovu et al. (2020), Uganda	High risk	High risk	High risk	High risk	Low risk	Low risk	High risk	71.4
Reshma Naik et al. (2015), South Africa	Low risk	Low risk	High risk	High risk	Low risk	Low risk	Low risk	28.6
Lucy Anne Parker et al. (2015), Swaziland	Low risk	Low risk	Low risk	Low risk	Low risk	Low risk	Low risk	00.0
K du Preez et al. (2020), South Africa	High risk	High risk	High risk	High risk	Low risk	Low risk	Low risk	57.1

### Eligibility criteria

Eligibility criteria for inclusion and exclusion were developed to identify original research that empirically evaluated or tested interventions/strategies linking young African people to healthcare. Articles were eligible for inclusion if they were (a) conducted in Africa, (b) described an intervention or strategy with outcomes linking young people to healthcare, (c) Interventions were specific to young people aged 10–24 or reported on young people aged 10–24 living in Africa., (d) were written in English, and (e) published between 2011 and 2021. Studies that included intervention designs ranging from non-experimental evaluations to quasi-experimental and randomized control trials were included and reviewed. Systematic reviews, qualitative studies, non-empirical studies (e.g., reviews, commentaries, editorials, and dissertations), and studies that did not explicitly assess links to healthcare among young people were excluded from the review.

### Data extraction

The data extraction for studies that met the inclusion criteria was as follows:

(1) title, author, country, study objective, and design (2); information about the intervention assessed, also including the type and impact of the intervention on linkages in healthcare for treatment and assessment of health, and targeted young people; (3) components of the intervention; and (4) RE-AIM framework implementation outcomes which included (a) reach (absolute number, proportion, and representativeness of young people in the intervention study), (b) efficacy (impact of the intervention on young people's ability to be linked to healthcare in a desired location, including social, economic factors and outcomes), (c) adoption (the absolute number, proportion, and settings participating in the intervention, as well as the extent to which the settings chosen are representative of settings used or visited by the target population), (d) implementation (the consistency of delivery as intended, time, and cost of implementation), and (e) maintenance (the degree to which a program has established standard operating procedure at the organizational level, or the program's long-term consequences on individual outcomes) (46).

### Data analysis

The data was analyzed using narrative synthesis, synthesizing the details of the RE-AIM reporting component. The reporting of RE-AIM dimensions was evaluated with a data extraction instrument created and validated (46). It contains elements of implementation outcome according to the RE-AIM framework (40). A meta-analysis was not included in this study because the study design and measurement results of the articles included in this review were non-uniform. The evaluation of each article was a customized RE-AIM data extraction form containing columns with the components of each RE-AIM dimension, and data extraction was done to evaluate all the interventions involved. This form (Supplementary File S1; Table 2) included a review of the general characteristics of the papers included in the analysis that was separately performed by three authors (TG, UN, CO) (40, 47). The adapted RE-AIM data extraction form is in Supplementary File S1. The data extraction form is used to compute percentages of interventions that met the criteria for the five RE-AIM dimensions (reach, efficacy, adoption, implementation, and maintenance). The RE-AIM components were summarized with frequencies, proportions, and means. The reported frequencies and proportion of the components for each study included in the review had their RE-AIM dimension, calculated separately in Table 3. Within the RE-AIM dimension, the average proportion (Table 3) calculated was across the 16 unique interventions included in the review. This analysis provided a comparable summary score (Table 3) across the interventions. The percentage and number of interventions in each RE-AIM dimension are shown in Table 3.

**Table 2:** Shows the selected study's study design and the number of interventions.

Number of interventions	Study, location	Design description
1.	Ahmed Saeed et al. (2017), Malawi	Quasi-experimental study. (Design not stated in paper) Testing of Household members(specifically children and young persons) of HIV-infected patients(Index cases) enrolled in HIV services who reported untested.
2.	Caroline E. Boeke et al. (2018)a Uganda	The descriptive study was nested with the quasi-experimental research below.
	Caroline E. Boeke et al. (2018)b Uganda	Quasi-experimental study. (Design not stated in paper) A proactive follow-up intervention to improve linkage and retention among people living with HIV in a pre-/post- study
3.	Shraddha Bajaria, Amon Exavery, Noreen Toroka and Ramadhani Abdul,(2021) Kenya	Longitudinal Study, from the USAID Kizazi Kipya project in 79 councils of Tanzania.
4.	James Ayieko et al. (2019), Kenya and Uganda	A community-based cluster randomized trial. A patient-centered, multicomponent linkage strategy in Kenya and Uganda's SEARCH “test-and-treat” trial.
5.	Lillian B. Brown et al. (2020), Kenya and Uganda	Community-based RCT The study involved 32 rural communities in western Kenya and eastern Uganda.
6.	Larry W. Chang et al. (2021), Uganda	A pragmatic cluster-randomized trial in a high-risk, the highly mobile fishing community. The study community was divided into 40contiguous randomly allocated clusters(20 inter-vention clusters)
7.	Augustine T. Choko et al. (2015), Malawi.	A prospective study nested within a cluster-randomized trial comparing health outcomes between 14 clusters randomized to HIVST and 14 clusters randomized to routine (facility-based) HTC.
8.	Nolwenn Conan et al. (2020), Malawi	A cross-sectional survey was conducted. They were using two-stage cluster sampling. Selected households were asked to participate.
9.	Cari Courtenay-Quirk et al. (2018), Tanzania	A modified stepped-wedge design The intervention was implemented in 12 TB clinics in Pwani, Tanzania. The clinics were selected by convenience within two geographical clusters (Cluster 1 comprised four clinics, and Cluster 2 comprised eight clinics) with similar catchment populations. Each cluster included at least one referral hospital (district or regional level), a health center, and several directly observed therapy (DOT) centers. RCT mixed method survey was used for the data collection.
10.	Batya Elul et al. (2017), Mozambique	In the cluster-randomized trial, ten primary health facilities in Maputo and Hambane Province were randomly assigned the intervention or standard of care.
11.	Hewett et al. (2016), Zambia	Randomized Control Trial(RCT) The study was initiated at seven health service sites. There were two types of study sites: entry point sites, where clients were recruited into the study and referral sites to which clients were referred for additional services. Each district had at least one of the three entry point services: HIV testing and counseling, family planning, and voluntary medical male circumcision, referral service sites included SFH-operated integrated service centers, public hospitals/clinics, and partner NGO-run service centers; all referral sites were mapped and located within walking distance of the entry point locations.
12.	Niklaus Daniel Labhardt et al. (2014), South Arica Lesotho	The study was an open-label, two-armed cluster-randomized trial conducted in two rural catchment areas. This trial compares home-based HIV counseling and testing (HB-HTC) to mobile clinic HTC (MC-HTC)
13.	Joseph.K.B. Matovu et al. (2020), Uganda	Cross-sectional study
14.	Reshma Naik et al. (2015), South Africa	It was part of a larger cluster randomized controlled trial of door-to-door HB-HCT called ‘‘Good Start,’’
15.	Lucy Anne Parker et al. (2015), Swaziland	Cross-sectional study
16.	K du Preez et al. (2020), South Africa	Prospective cohort study

**Table 3:** Proportion of interventions reporting RE-AIM dimensions and components.

RE-AIM dimensions and components	Reporting frequency (*n* = 16)	Reporting proportion (%)
Reach		
Method to identify the target population	14	93.3%
Inclusion criteria	13	86.7%
Exclusion criteria	3	20.0%
Sample size	13	86.7%
Participation rate	1	6.7%
Characteristics of participants	15	100.0%
Characteristics of non-participants	4	26.7%
Representativeness	14	93.3%
Average of overall reach dimensions^a^	9.6	64.17%
Efficacy		
Measures/results for at least one follow-up	14	93.3%
Intent to treat utilized	7	46.7%
Quality-of-life measure	3	20.0%
Baseline activity measured	15	100.0%
Percent attrition	3	20.0%
Average of overall efficacy dimensions^a^	8.4	56.0%
Adoption		
Description of intervention location	15	100%
Description of staff who delivered intervention	15	100%
Method to identify target delivery agent	12	80.0%
Level of expertise of a delivery agent	3	20.0%
Adoption rate	0	0.0%
Average of overall adoption dimensions^a^	9.0	60.0%
Implementation		
Intervention duration and frequency	15	100.0%
Extent protocol delivered as intended	5	33.3%
Measures of cost of implementation	4	26.7%
Average implementation dimensions^a^	8.0	53.3%
Maintenance		
Individual-level maintenance		
Was individual behavior assessed ≥6 months post-intervention	12	80.0%
Was individual behavior assessed ≥24 months post-intervention	4	26.7%
Was individual behavior assessed ≥48 months post-intervention	0	0.0%
Program-level maintenance		
Indicators of program continuation	0	0.0%
Some measure/discussion of alignment with organization/setting	4	26.7%
Measures of cost of maintenance	0	00.0%
Average of overall maintenance dimensions^a^	3.5	23.3%

Components were included to ensure relevance with Linkage to care in young people.

^a^ Average percent for overall 26 components within each RE-AIM dimension. The proportions are based on the 16 unique interventions included in the Review.

### Risk of bias

The interventions were compared systematically using the Cochrane Collaboration risk-of-bias tool to evaluate the rigor of each intervention (48). The tool has six domains: selection bias, performance bias, detection bias, attrition bias, reporting bias, and other sources of bias (48). Using the guideline for each domain, three writers (TG, UN, CO) independently rated the risk of bias as low, high, or uncertain. The raters discussed each domain of the evaluation tool to ensure accuracy and consistent judgment. If the ratings differed, reasons for the differences were addressed and re-evaluated to obtain a consensus. The Cochrane Collaboration developed the risk of bias assessment instrument to examine the interventions’ internal validity of the interventions included in the review; no study was removed because it risked a biased score (Table 1).

## Results

2,383 potentially relevant citations were obtained after an initial database search.224 titles and abstracts were then screened (Figure 1). From these citations, 224 papers were included to complete a full-text review, and 94 were excluded (Figure 1). The most frequently cited reasons for exclusions were:

1) Studies did not include links to healthcare in young people (10–24years) (*n* = 39),
2) Studies did not include young people aged 10–24 years or have young people within the 10–24 years in the analysis of their results (*n* = 33), and
3) Studies did not meet the study design criteria (*n* = 20).

### Features of the included studies

Seventeen articles pulled from electronic data searches were retained in the final group. Table 2 shows the interventions and study designs of the final included articles. Identified in our report were 16 interventions (summary of interventions shown in Table 4) from the 17 publications presented The interventions in the articles were performed between 2011 and 2021. Twelve were in East Africa, and four were in South Africa. Most of the young people targeted were between 15 and 24 years old. Out of the seventeen studies, nine (53%) were randomized controlled trials, three (18%) were quasi-experimental designs, and five (29%) were observational studies.

**Table 4:** Summarizes all interventions in the studies but does not show how the interventions were combined in all the studies.

Home-based counseling and testing	Facility counseling and testing	Follow-up phone calls and home visits enhanced counseling	Referrals to counseling, testing, and care	Mobile clinic HTC^a^ and homebased HTC^a^	Incentive reimbursement of transportation	Escorts	Short message service	Community health campaigns	Social network	HIVST^b^	Home visits enhanced counseling	Homebased HTC^a^	Referral logbook and re-linkage to care
Ahmed Saeed et al. (2017), Lillian B. Brown et al. (2020)	Ahmed Saeed et al. (2017)	Caroline E. Boeke et al. (2018)a, Caroline E. Boeke et al. (2018)b, Shraddha Bajaria, Amon Exavery, Noreen Toroka and Ramadhani Abdul (2021), James Ayieko et al. (2019), Larry W. Chang et al. (2020), Nolwenn Conan et al. (2020), Niklaus Daniel Labhardt et al. (2014), Lucy Anne Parker et al. (2015)	Larry W.Chang et al. (2020), Shraddha Bajaria, Amon Exavery, Noreen Toroka and Ramadhani Abdul (2021), Augustine T. Choko et al. (2015), Hewett et al. (2016), Cari Courtenay-Quirk et al. (2018)	Lucy Anne Parker et al. (2015)	James Ayieko et al. (2019), Batya Elul et al. (2017), Joseph.K.B. Matovu et al. (2020)	Hewett et al. (2016)	Batya Elul et al. (2017)	Lillian B. Brown et al. (2020)	Lillian B. Brown et al. (2020)	Choko et al. (2015), Joseph.K.B. Matovu et al. (2020)	Larry W. Chang et al. (2020), Nolwenn Conan et al. (2020)	Niklaus Daniel Labhardt et al. (2014)	Cari Courtenay-Quirk et al. (2018)

^a^ HIV counseling and testing (HTC).

^b^ HIV Self-testing (HIVST).

A detailed description of the strategies for linkage and how they differ from health services can be seen in the Supplementary File S1 (Table 2). Four studies targeted individuals <25 years (49–52). Seventeen studies targeted linkage to care for HIV treatment among the study population (44, 45, 50, 51, 53–63). In addition to HIV linkage to care, two of the studies also had links to healthcare for sexual and reproductive health services (HIV, family planning, and male circumcision (55, 56, 63). Two of the studies had links to healthcare for tuberculosis treatment (49).

### Quality of evidence

Quality assessment of the selected articles was reported in Table 1, showing the level of bias risk among the interventions, which varied from no risk of bias to 71.4%. Two interventions using quantitative methods, for their interventions, were found to have a 0.0% (low) risk of bias (58, 59). The risk of bias for quantitative methods ranged from 0.0% (low) (58, 59) to 71.4% (high) (65). The mixed-method interventions had an increased risk of bias, 57.1% (59), to extremely high, 71.4% (57). The most common strengths among the interventions were conducting a longitudinal follow-up of study participants over time, selecting and assigning participants, and descriptive reporting of the intervention. Weaknesses observed by the majority of the studies were the lack of acknowledgment of the participation rate (44, 45, 50, 51, 54–63), description of the non-participant (44, 45, 50, 51, 53–61), and the quality of life (44, 45, 50, 51, 53–63). Attrition was mentioned in two studies but not analyzed (45, 54); only one study examined the attrition rate (66).

### Reporting of Re-AIM dimensions

The average reporting rates (defined here as the overall percentage of components) across all the interventions were highest for reach with 9.6 (64.2%), followed by adoption 9.0 (60.0%), followed by efficacy 8.4 (56.0%) and), with lowest for implementation 8 (53.3%) and maintenance 4 (26.7%). A summary of the overall percentages of interventions reported under each RE-AIM dimension can be seen in Table 3; it shows scores within each component across the RE-AIM framework.

### Reach

Reach was the most consistently reported RE-AIM dimension across all interventions (64.2%). In Table 3, the average reach component reported was 9.6 (64.2%). The characteristics of study participants were the most frequently reported 15 (100%). All interventions except three were reported on sample size (45, 54, 66), defined as the number of participants who consented to participate in the study/intervention.

Only three studies focused on recruitment and strategies among the interventions, mainly for young people. They had as objectives the linkage of young people to healthcare (50, 51, 66), while one focused on children <13 linked to healthcare (49). The sample size in these studies ranged from *n* = 711 (51) to *n* = 14538 (50). The other studies that included young people but did not explicitly focus on them had sample sizes for young people ranging from *n* = 152 to *n* = 1894 (45, 47, 53–61, 63). Three studies did not explicitly mention sample size. One paper inferred the study population since the study was nested in another study (46, 54). The other paper did not indicate this population total (66). Inclusion criteria were mentioned in 13(86.7%) of the interventions. These criteria included the age of participants, place of residence, treatment facility, and community of first contact. They confirmed the diagnosis of a particular disease or health condition (HIV, TB, or sexual health needs).

Reported participant characteristics included age, gender, educational level, marital status, socioeconomic status, disease type (reported as those who were HIV positive or TB positive), and the number of diagnoses of disease (those confirmed with either HIV or TB). Methods used to identify the target population varied across interventions, from single-sentence descriptors to detailed protocol reporting (45, 47, 50, 51, 53–63). Identification of the target population was the next most reported reach component. Twelve (80%) of the interventions were conducted in eastern Africa; all the interventions addressed linkage to care for young people in preventing and treating HIV (45, 50, 51, 53–57, 59, 61–63).

Strategies to identify the targeted population included using community medical outreaches, community stakeholders, and social networking through surveys of routinely collected data, family members, and community census. These studies did not report whether these identification methods facilitated or hindered their ability to reach the targeted population. Only one intervention reported a participation rate (53). About 14 (93.3%) of the interventions reported representativeness of recruitment of study participants. These parameters were compared with the population target by the study participants (e.g., age, level of education), which enabled the researcher to evaluate the degree to which the intervention might be generalized across the target population and the component's environment. As a measure of representativeness, the rigor of the study design was reported. RCTs reported this study design as one of their strengths in the representativeness of their interventions.

Four interventions (26.7%) gave data that described the non-participants compared to participants of the targeted population in their studies. A few reasons for non-participation indicated those who did not test positive for the targeted disease, deaths, lack of communication, and dissolution of marriages.

### Efficacy

Seven interventions (46.7%) inferred intention-to-treat utilization in their studies. However, it was not explicitly stated, while the other studies analyzed only data from participants who completed the intervention. All interventions included in the review included links to healthcare as primary outcomes. HIV linkage to care included those linked within 1 week (49, 51), one month (45, 47, 55, 58–60), 3 months (55, 59), 6 months (58, 62, 66) and 1 year (50). All 16 Interventions were used to cut across various studies, as some used multiple strategies. How the strategies were combined and accessed differed for all 16 tested interventions. These included the following: facility counseling and testing (51); home-based counseling; and testing only (51, 58, 61–63, 67), enhanced counseling (49, 61, 63, 67), follow-up phone calls; and home visits enhanced counseling (44, 50, 54, 59), referrals including the use of a referral logbook; and referrals made for re-linkage made for counseling; testing and care (49, 50, 55, 56, 59, 63), mobile clinic (58, 59), incentive reimbursement of transportation (45, 52, 53), escort (56), short message service (SMS) (53), community health campaigns (62), social network (62) and using HIVST (52, 55).

Three interventions (20.0%) reported attrition (44, 54, 56, 59), but only one calculated the percentage of attrition (56). Attrition rates were accessed with loss to follow-up of participants and non-use of the intervention (56). Causes for attrition included participants’ relocation, death, difficulty in communication (not owning a phone), family breakup, and religious issues.

### Adoption

The average proportion reporting on adoption components was 9 (60.0%). The most well-described element in this aspect was the description of the intervention location and the description of staff who delivered intervention 15 (100%). The location descriptions were very detailed, including homes, community centers or gatherings, and health facilities. Most of the interventions were restricted to a specific geographical area. Some interventions occurred within one country (44, 45, 52, 54, 60, 62, 63). The staff who delivered the interventions had varying levels of expertise and included research assistants, community leaders, and study staff. The exact level of knowledge of the delivery agent was reported in three interventions (53, 55, 61). The study staff that delivered interventions was identified through their participation in the research project or their role in their current job that co-existed with the study. The staff responsibilities ranged from offering the intervention through, counseling, testing, providing cash incentives, training participants, and issuing referrals to follow-up on referrals. Surprisingly, none of the interventions reported an adoption rate in their studies.

### Implementation

The average percentage of respondents reported implementation components was 53.3% (8 interventions). All 16(100.0%) interventions reported the design of the intervention. They provided information on intervention duration and frequency (44, 45, 50, 51, 53–63). Intervention varied in duration from a single session to two or more sessions. Five (33.3%) of the interventions explicitly reported fidelity and the extent to which the intervention protocol was delivered as intended (44, 55, 56, 59, 63). A cost analysis was not part of the initial trial protocol in one intervention (59). Four (26.7%) studies mentioned the interventions’ cost (49, 55, 56, 59).

Costs were reported in various ways:

• The actual expenditures made for each campaign component (59).
• The cost-effectiveness for illnesses (HIV/AIDS and cervical cancer were high in the enhanced service model) (56).
• Comparison of per-episode costs of providing interventions (e.g., comparison of the costs of HIVST to the costs of facility-based testing) made in some studies (44, 53, 54, 56, 58).

However, one did not report it but indicated that this was to be declared fully elsewhere (55, 58). In another report, the cost was from the service provider's perspective (58).

### Maintenance

The average number reported on maintenance components was about 3.5 (23.3%), the lowest average in all the dimensions described. The highest registered maintenance components are the individual-level indicators reported more frequently than program-level indicators. Seven (46.7%) interventions reported at least one follow-up measure, particularly the primary outcomes at 6 months (44, 45, 51, 54, 55, 56, 58, 59). Some interventions had a follow-up of fewer than 6 months (51, 55, 59). The most extended follow-up period reported was 24 months after baseline assessment (55, 62, 63). However, a few interventions had follow-up assessments beyond 24 months after intervention completion: for 36 months (62, 63). One intervention was reported (44, 68) on program-level maintenance or sustainability indicators. None of the interventions explicitly stated that the interventions were sustained beyond the study period. One article noted the intervention might continue because of the low cost (44, 68). Four (26.7) of the interventions discussed the alignment with the organization. None of the interventions showed any measures of the cost of maintenance. They included interventions reported on 20 (76.92%) of the 26 components of the RE-AIM dimensions.

## Discussion

This review systematically evaluates interventions linking young people in Africa to healthcare. This review goes beyond assessing the effectiveness of the interventions and reports on implementation results that follow the concepts of the RE-AIM framework. The RE-AIM framework was used to determine the impact of these interventions linking young people to healthcare. The five components of the RE-AIM framework are vital for converting research evidence to practice: reach, effectiveness, adoption, implementation, and maintenance (35, 47). These components play a significant role in understanding the factors that impact linkages in healthcare in various elements but also assess the vital missing aspects in these interventions needed for future sustainability and adaptation by the communities where the young people participate.

In 17 studies, 16 interventions linking young people to healthcare were identified, described, and evaluated based on the five RE-AIM dimensions.

This study showed that most interventions focused on HIV treatment/prevention services. Surprisingly, other health conditions focused on were all linked with HIV services, which leaves the question of whether other health conditions are less prioritized when connecting young people to healthcare. The funding agencies in research dictate the role of focus in research for young people in Africa (69, 70). Conflicts between funding and policy actors over how society will benefit from research usually occur (69). Analysts have observed that investments in innovation and science may not always result in social and economic progress (71–73).

At some level, all RE-AIM dimensions were reported in the 16 interventions, but implementation and maintenance were the least developed dimensions. Most interventions are based on specific components, such as identifying the target population, intervention location, intervention duration, and frequency. Still, they left out the broader areas, such as the program-level sustainability and implementation costs. However, this is not unique to this study, as other systematic reviews using the RE-AIM framework have also shown limited reporting of these dimensions (35, 46, 74). It shows that there is more focus on the intervention effectiveness and less emphasis on factors that may impact the translation of these effective interventions to the practical everyday setting. Researchers must focus on these dimensions to understand how these findings can be applied to the communities and populations and how the available resources can be used to implement these findings on a larger scale (35, 39). Ultimately, these interventions can improve the health of young people in Africa.

It is vital to know how to reach the targeted population and what factors would influence positive changes in behavior to make the interventions effective. It informs decisions to take towards future scale-up and dissemination of interventions. Some interventions that combined a mobile hybrid community-based testing strategy with a novel patient-centered, multicomponent linkage strategy resulted in high linkage rates, with half of all individuals in need of HIV care linked within one week of HIV testing and three-quarters linked within a year (45, 58, 61, 67). This review method described participants’ characteristics, sample sizes, and representativeness in interventions to identify the target population. On the other hand, the interventions rarely described the attributes of non-participants and did not give reasons for non-participation. Few selected studies showed information about the external population from which the study sample was obtained (59, 62, 63). Knowing the background of the population would give a deeper understanding of factors that may influence the participation of participants in these interventions. It would also help the researchers amplify these interventions to the general population and beyond.

Unlike other reviews that have reported effectiveness, effectiveness was not the most stated RE-AIM element in this review. Divergent views have been seen in past reviews, where intervention effectiveness was the most reported RE-AIM element across all interventions (76, 77). Findings from this systematic review highlight the impact of the interventions on linking young people to healthcare, with about 56% of the interventions reporting improvements in connecting participants to healthcare. Only seven described intent-to-treat analyses, though, in four, it was mentioned but not seen in the analysis; this may have positively influenced the interventions and affected the participants involved in the intervention to the follow-up stage. Only three studies provided information on attrition. In one study, high attrition of participants was observed between receiving a positive HIV test result and linkage to healthcare (59). In another study, it was attributed that they focused on poorly performing facilities. So, their findings may not be generalizable to sites as they had much lower attrition. The strengths of this study focused on including only struggling health facilities that could more readily identify gaps and areas for improvement (44). Death was another reason for attrition (53). Accounting for attrition is crucial in detecting those factors that are barriers to links to healthcare in young people.

In the RE-AIM component for adoption descriptions on intervention location, staff who delivered the interventions and identified the target delivery agent were well documented. Nevertheless, there was no mention of the level of expertise of the delivery agent and the adoption rate; this was consistent, observed to be under-reported in the methods used to enhance the adoption rate in other reviews that used RE-AIM (76). None of the studies reported an adoption rate, so we cannot identify the factors that promote adoption across the various settings.

Many of the implementation components in this review were poorly reported, especially the cost of the interventions, which would help determine how feasible these interventions can be implemented in real-world settings. Only four studies reported the cost and cost-effectiveness of implementing interventions (44, 54–56). None of the interventions reported fidelity to the intervention. Studies should report on this as this would, in the future, enhance the capacity for interventions to be translated to other settings.

Almost a quarter of the maintenance interventions were reported on this RE-AIM dimension. Compared with other reviews done in this component, this is a better outcome when assessed with reviews that described maintenance between 0.0% and 11.0% (46, 47). Most of the interventions measured maintenance of individual behavior at least 6 months following the completion of the intervention, while four measured 24 months but none at 48 months. The review showed that most interventions failed to understand the long-term maintenance of intervention participants’ behavioral changes and the sustainability of the interventions at the implementation site. Individual and program-level characteristics and broader socio-cultural and community-level ones play a role in the long-term impact of intervention (46).

### Strengths and limitations

The strengths of this study are. First, data was obtained using a well-organized, systematic, and well-designed search strategy that was created with the help of a Cochrane search expert and supplemented by a manual search in the bibliography list of included articles. Second, as far as we know, this is the first study to examine collated data measurements for implementation outcomes among studies on linking young people to healthcare in Africa using the RE-AIM framework.

One limitation observed in this study is that we did not do a meta-analysis. However, this is not the focus of this systematic review; a meta-analysis would not have supported the heterogeneity of the interventions involved in this study. Selection bias could have occurred in our search strategy since we limited our search to all published articles in English. Given the limitation of the risks of Cochrane collaboration bias assessment tools, the tool used to assess the internal validity of the interventions included in this review was not used to select articles to have. The reports generated in this review were based on how the chosen interventions reported certain aspects of the RE-AIM framework. Some of the interventions did not state other ailments affecting young people. They also did not report on the accessibility of healthcare, staff attitude, communication, medical competency, guideline-driven care, and age-appropriate environments. This would make it difficult to assess if these interventions are universally appropriate and acceptable for other ailments apart from HIV (4). Also observed was that most of the interventions did not include health outcomes. It would be difficult to assess if the interventions should be generalized.

### Implication

Future studies should specifically evaluate the effectiveness of young people's health interventions in these settings. Further studies with longer-term follow-ups are required, and study authors should use standardized and validated measurement instruments to maximize the comparability of results. These research studies should assess how to bridge the gap between practice and research in young people's care.

More research with extended follow-up periods is needed to optimize the comparability of outcomes. Study authors should also use standardized and verified assessment tools. Future research studies should evaluate interventions’ impact on research, practice, and policy. It would be vital to conduct research with experts in young people's health to prioritize research gaps and suggest immediate action areas. Young people's health is still a developing field with many unmet needs. Additionally, this exercise can give donors a thorough understanding of the predicted value and viability of investing in these research gaps and assessing additional gaps in the evidence for young people's health.

## Conclusion

In conclusion, timely linking to healthcare remains critical in treating and preventing ailments, especially HIV. Evidence seen in this review indicates that interventions created to connect young people to care, especially in HIV, can help link them to healthcare and strengthen the programs by giving more access to young people who are HIV positive. Since implementation factors and RE-AIM components are vital in assessing the impact of interventions in linking to healthcare, more research is needed to determine other health ailments. Also, further research is required to show outcome indicators of interventions that target young people. More emphasis should be placed on the adoption, implementation, and maintenance/sustainability of these interventions in Africa. Beyond this, it is essential to note that this systematic review highlights the importance of healthcare linkages among young people in Africa and will help disseminate findings from the interventions. These findings can review linkage strategies performed among young people in Africa.

## Data Availability

The original contributions presented in the study are included in the article/Supplementary Material, further inquiries can be directed to the corresponding author.
