# Supplementary material for: Interventions connecting young people living in Africa to healthcare; a systematic review using the RE-AIM framework
Source: Front Health Serv. 2024 Jan 31;4:1140699. doi: 10.3389/frhs.2024.1140699 (PMC10864512; doi:10.3389/frhs.2024.1140699)
Supplement: Supplementary file 1 [file Table1.docx]

| Study,Location | Design Description | Outcome of Interest | RE-AIM DIMENSION | | | | |
| --- | --- | --- | --- | --- | --- | --- | --- |
|  |  |  | Reach | Adoption | Implementation | Efficacy/Effectiveness | Maintenance |
| Ahmed Saeed *et al* (2017),Malawi | Household members of HIV‐infected patients(Index cases) enrolled in HIV services who reported untested | identify and link HIV-infected children (1-15 years) and young persons (>15-24 years) to care | 711 children and young persons were tested and newly diagnosed 30 HIV-infected persons identified. | Community health worker (CHW) performed the tests and follow-up at home and  facility-based. | home‐ or facility‐based HIV testing and counselling (HTC) of the index cases household  The household HIV testing and counselling (HH-HTC) programme was aimed at identifying and linking HIV-infected children and young persons to care.  After screening, any patient reporting a household member with an unknown HIV status was offered the option of either home- or facility-based HH-HTC for their untested household members. | Of the 30 HIV-infected persons identified, 23 (76.6%) were linked to HIV services; 18 of the 20 eligible for ART (90.0%) were initiated.  Median time (IQR) from identification to enrolment into HIV services was 4 days (1–8) and from  identification to ART start was 6 days (1–8). | All persons newly diagnosed with HIV infection and  those who were identified with a known HIV infection but  not enrolled in HIV care were followed for up to three  months by CHWs. There was With a 93%  acceptance rate of household testing. Strategy was found to be feasible and facilitates the identification  and timely linkage to care of HIV-infected children and young persons |
| Caroline E. Boeke et al (2018) Uganda | Comparative study. They compared patient linkage to and retention  in HIV care in the 9 months before implementation of the intervention to the 9 months after implementation.  linkage to care (defined as registering for pre-ART or ART care at the facility within  1 month of HIV diagnosis) and 6-month retention in care (having a visit 3-6 months after ART initiation) | Increase in proactive patient follow-up and enhanced counselling would improve patient linkage and retention | 152(15.2%) children and young people less than 18 years. Those 10-18(80(8.6%) had 20.9% male. It was skewed | Lay Health workers in the health facilities | They implemented a quality of care improvement intervention  package that included training Including ; best practices for patient follow-up and counseling, including  improved appointment recordkeeping, phone calls and home visits to lost patients, and enhanced adherence  counseling strategies; and strengthening oversight of these processes | No significant impact was seen in improved patient linkage to care. Poor linkage to care was observed in adolescents (ages 10-18 years) compared to adults  (ages 19-48 years). . In particular,  adolescent females had the lowest linkage (35.5%) of any  sex/age category combination.  60.0% of the children  < 10 years and 55.4% of adolescents 10–18 years were being tested for the first time. 3.4% of patients were known  tuberculosis (TB) positive. There was a greater increase in linkage among smaller facilities/programs:  level III facilities (13.4%) compared to level IV facilities  (− 1.6%; effect estimate: 14.2, 95% CI: 0.5–27.9%; p =  0.04); facilities with fewer expert clients (< 5: 11.5%; 5+:  − 4.4%; effect estimate: 14.3, 95% CI: 3.3–28.3%; p =  0.045); and facilities with fewer adult ART days per week  (1 day: 16.7%; 2+ days: − 4.3%; effect estimate: 17.4, 95%  CI: 3.9–30.9%; p = 0.01). There was also a greater increase in linkage among facilities reporting issues with  phone funding at baseline (16.0%) compared to those  who did not report this as an issue (− 0.1%; effect estimate: 15.8, 95% CI: 1.8–29.8%; p = 0.03).  The annual cost per additional patient retained in care was estimated to be $47, dropping to $32 if data from the one-month intervention | Individual level :  HIV positive Participants had follow-up at least three times within 6 months  Program level:  The authors implied that due to the success of the intervention inbpaediatrics and adolescents and its low cost there is possibility that this approach will be scaled –up.  these types of relatively low-cost interventions should be emphasized as a strategy to reduce the burden of HIV |
| Caroline E. Boeke et al (2018) Uganda | Descriptive study | Assessment of the patterns of engagement in care in 20 Ugandan health facilities with low retention based on national reporting | Total number of participants newly diagnosed HIV positive and assessed for linkage were 928. | professional data collectors and senior study staff using Surv | an intervention to promote proactive follow-up and counseling practices. | Participants 10-18 years were 78 in number i.e 39.7% of the total participants linked to care in a month)  was poorest in adolescents (ages 10-18 years) compared to adults (ages 19-48 years) (39.7% vs. 53.5%; p = 0.03). In particular, adolescent females had the lowest linkage (35.5%) of any sex/age category combination. | none |
| Shraddha Bajaria, Amon Exavery  , Noreen Toroka and Ramadhani Abdul(2021) kenya | Longitudinal Study, from the USAID Kizazi Kipya project in 79 councils of Tanzania. | the magnitude of caregivers’ disability and  assesses its relationship with successful linkage to care of their OVC living with HIV/AIDS. in Tanzania | 14,538 HIV positive orphans and vulnerable  children (OVCs) aged 0–19 years | Caregivers living with HIV positive OVCs. | Referrals to counseling and testing services and care and treatment clinics  (CTCs). | OVCs living with disabled caregivers were less likely to be linked to care (OR 0.76, 95% CI 0.58,  0.99). OVC living with a HIV positive caregivers (OR 1.25, 95% CI 1.12, 1.41). OVC living in household with high  socio-economic status were less likely to be linked to care (OR 0.76, 95% CI 0.67, 0.86) than those in low-SES  households. | Individual level :  Participants had follow -up but length of follow-up not known though study was for 12 months  Program level:  No Indicators of program level maintenance were not reported  Follow-up linkage rates for the younger was not reported but for the whole study . multicomponent strategy linked half of all HIV-positive persons who were not in care to HIV care within 1 week of testing and by 1 year 3 quarters of all individuals had linked. Caregivers were followed up by community workers who gave support by visiting beneficiaries on a monthly basis and following up on the referrals and services needed or  provided |
| Oluwafemi Atanda Adeagbo et al(2019), South Africa  Remove !!! | A cluster randomised controlled trial (cRCT) | increase knowledge of HIV status and improve linkage to HIV care or prevention services such as PrEP among young women aged 18 to 24 years.  to determine the effect of peer delivery HIV self-testing to support linkage to HIV prevention among young women | 12 000 young  people aged 18 to 30 years | Twenty- four pairs of peer navigators | (1) incentivised-peer-networks: peernavigators recruited participants ‘seeds’ to distribute up to  five HIVST packs and HIV prevention information to peers  within their social networks. Seeds receive an incentive  (20 Rand = US$1.5) for each respondent who contacts a  peer-navigator for additional HIVST packs to distribute;  (2) peer-navigator-distribution: peer-navigators distribute  HIVST packs and information directly to young people;  (3) standard of care: peer-navigators distribute referral  slips and information. All arms promote sexual health  information and provide barcoded clinic referral slips  to facilitate linkage to HIV testing, prevention and care  services |  |  |
| James Ayieko et al (2019), kenya and Uganda | a community-based cluster randomized trial | Increase in the proportion of the population linked to care  within 1 year while evaluating the factors associated with linkage at 7, 30,  and 365 days after diagnosis. | 395 AYA 15-24 years were involved in this study | 1. clinic staff were introduced to in person participants who tested at a health campaign, 2. Using a study-provided phone for participants who tested at home or other location. | After population-based, community-wide HIV testing, eligible participants were (1) introduced to clinic staff after testing, (2) provided a telephone “hot-line”  for enquiries, (3) provided an appointment reminder phone call, (4)  given transport reimbursement on linkage, and (5) tracked if linkage  appointment was missed.  in conclusion, timely linkage to care remains critical in treatment and prevention of HIV. Combining a mobile hybrid community-based testing strategy with a novel patientcentered, multicomponent linkage strategy resulted in high linkage rates, with half of all individuals in need of HIV care linked within 1 week of HIV testing and three-quarters linked within a year | Individuals who  were younger [adjusted Risk Ratio (aRR) 0.83,  95% confidence interval (CI): 0.74 to 0.94], tested at home vs. community campaign (aRR = 0.87, 95% CI: 0.81 to 0.94), had a high HIV-risk vs. low-risk occupation (aRR = 0.81, 95% CI: 0.75 to 0.88), and were wealthier (aRR 0.90, 95% CI: 0.83 to 0.97) were less likely to link. Linkage did not differ by marital status, stable  residence, level of education, or having a phone contact. Age and occupation were associated with linkage to care. | Individual level :  Participants had follow -up for 12 months  Program level:  No Indicators of program level maintenance were not reported  Follow-up linkage rates for the younger was not reported but for the whole study . multicomponent strategy linked half of all HIV-positive persons who were not in care to HIV care within 1 week of testing and by 1 year 3 quarters of all individuals had linked. |
| Lillian B. Brown et al (2020), Kenya and Uganda | Community- based RCT the study involved 32 rural communities in western Kenya and eastern Uganda | Youth whose immediate social network included contacts who were also HIV-infected would be more likely to initiate ART than those without HIV-infected contacts, and that the association would be stronger for contacts who were ART-experienced, of the same age, and who were named multiple times as sources of social support (strong ties). | 1,120 HIV-infected youth 15-24 years who were ART-naïve. | HIV positive youth who is on ART treatment(Peer Support)  Field staff collected data on tablets | Census enumeration was followed by HIV and multidisease testing using a hybrid model that combined multidisease community health campaigns with home-based testing for nonattendees, and which reached 90% of the populationUse of social network to link HIV positive youth(15-24 years) to care | 270 (33.5%) named at least one baseline HIV-infected contact; 70% (569/805) subsequently initiated ART. Youth with ≥1 HIV-infected same-age baseline contact were more likely to initiate ART (aOR 2.95; 95% CI 1.49–5.86) than those with no HIV-infected contact, particularly if the contact was a strong tie (named in > 1 domain; aOR 5.33; 95% CI 3.34 – 8.52).HIV- infected same age contact who was a strong tie remained associated with ART initiation (aOR 2.81; 95% CI 1.76 – 4.49). Approximately 70% (569/805) initiated ART before follow-up year three  Peer support also plays an important role in initial linkage to care and ART initiation. | Individual level :  Follow-up at 6, 12 and 36 months  Program level:  No Indicators of program level maintenance were not reported |
| Larry W.Chang et al (2020), Uganda | A pragmatic cluster-randomized  trial in a high-risk,  highly mobile fishing community  The study community was divided into 40contiguous ,randomly allocated clusters(20inter-vention clusters, n= 1,054 participants at baseline;20 control clusters,n= 1,094 participants at baseline) | Primary outcome  Increased self-reported  linkage to HIV care  ,antiretroviral therapy(ART)use,  and male circumcision  and the primary biologic outcome of HIV viral suppression (<400copies/mL).  Secondary outcomes  HIV testing coverage, HIV incidence, and consistent condom use | A total of 549 Youth(15-24years), that is 52% of the study population | Community health workers used the strategy called “Health Scouts” | The Health Scout intervention included   1. Counselling intervention was   designed to promote relevant  Tailored information, motivation,  and behavioural skills to improve client  engagement in HIV treatment and prevention services.   1. Health Scout phone application. Where functioned as a decision and counselling support mobile health   Core components of the intervention included (1) CHW (Health Scout)-based delivery; (2) counseling using motivational interviewinginformed strategies; (3) household-based delivery; and (4) mobile health-supp | Intention-to-treat analysis was found  higher HIV care coverage (PRR: 1.06, 95% CI: 1.01 to 1.10,  p= 0.011) and ART coverage (PRR:1.05 , 95% CI:1.01 to 1.10,p= 0.028) among HIV–positive participants in the intervention compared with the control arm. Male circumcision coverage among all men (PRR:1.05, 95% CI:0.96to 1.14,p= 0.31) and HIV viral . | Individual level :  Follow-up from 3  months and thereafter  every 3 months till month 36.  Program level:  No Indicators of program  level maintenance were not reported.  A novel community  Health worker  intervention improved  HIV care and ART coverage  in  an HIV hyperendemic setting but did not clearly improve male circumcision coverage or HIV viral suppression. |
| Jenala Chipungu et al (2017), Zambia | A representative cross sectional survey. This study used a convergent design, including concurrent implementation of qualitative and quantitative methods a | Increase in the intention to link to care amongst potential HIVST users and the suitability of three linkage to care strategies | A total of 571 are aged 16-24 years that is 35% of the population | Survey interviewers | Linkage to care strategies—text message, phone call and home visits were collected. | In this age-group 487(85%) had an Intent to link to care within a week if self-testing result is positive, while 307(54%) preferred follow-up home visit strategy. These were found to be not significant although this age-group was not specifically reported on for associations.  Prior testing for HIV and low-income status had increased odds of intention to link to care. Home visits and phone calls are preferred follow-up methods for those that would not link to care after receiving a positive result from an HIVST. These findings are important for the development of HIVST policy guidelines and implementation strategies for linkage to care | Individual level :  No follow-up done.  Program level:  No Indicators of program level maintenance were not reported |
| Augustine T. Choko et al(2015), Malawi. | This study was a prospective study nested within a cluster-randomised trial (ISRCTN02004005) comparing health outcomes between 14 clusters randomised to HIVST and 14 clusters randomised to routine (facility-based) HTC | To evaluate the uptake of testing, accuracy, linkage into care, and health outcomes when highly convenient and flexible but supported access to HIVST kits was provided to a well-defined and closely monitored population. | 1470 of study participant) young people Age 16-19 years participated in the study. | Trained resident volunteet-counsellors offered oral HIVST kits (OraQuick ADVANCE Rapid HIV-1/2 Antibody Test) to study participants and reported community events, with all deaths investigated by verbal autopsy.  Study Nurse | All the participants received instructions on how to use the kits, pre and post-counselling, and, for participants self-testing HIV positive, a referral card to attend an HIV care clinic. | Compared to months 1–12, the second year saw higher proportions  adolescents (24.7% versus 22.2%; p < 0.001),  HIVST uptake was more rapid in the second year than in the first year and was high among men and adolescents, two hard-to-reach populations.  In this study there was uncertainty about estimates of uptake and linkage to care as some apects of the study design may limit the accuracy of these results | Individual level :  24 month follow-up was done.  Program level:  No Indicators of program level maintenance were not reported |
| Nolwenn Conan et al (2020), Malawi | A cross-sectional survey was conducted between September 2016 and January 2017. Using two-stage cluster sampling, eligible adult individuals aged ≥15 years living in the selected households were asked to participate. | A population-based household survey was implemented to provide information on HIV prevalence and cascade of care to inform and prioritize community-based HIV interventions in the district. | 1475 young people aged which is 30.4% of the total study population | Existing Survey teams and Me´decins sans Frontières(MSF) | Participants were interviewed and tested for HIV at home. All participants tested HIV-positive at the time of the survey were advised to report their HIV test result to the health facility of their choice that MSF was supported in the district. | This was not analysed with the age group or if it was it was not reported. Evaluating the strength of the linkage to care, but Linkage to care was 78.0% (95%CI: 74.3–81.2) for all study participants | Individual level :  No follow-up done.  Program level:  No Indicators of program level maintenance were not reported |
| Cari Courtenay-Quirk et al(2018),Tanzania | a modified stepped-wedge desig  The intervention was implemented in 12 TB clinics in Pwani, Tanzania. The clinics were selected by convenience within two geographical clusters (Cluster 1 comprised four clinics and Cluster 2 comprised eight clinics) with similar catchment populations. Each cluster included at least one referral hospital (district or regional level), a health center, and several directly observed therapy (DOT) centers RCT | the feasibility of delivering an intervention for clinic-based a couples HIV testing and counseling (CHTC) strategy and linkage to care (LTC) for TB patients and their sexual partners in Tanzanian TB clinic settings  d an intervention to increase HIV testing and linkage to care (LTC) of newly diagnosed persons and re-linkage for TB/HIV patients | HTS Participants Younger than 15 years were 74 in number which is (5.8%) of the study while those 15–24 years were 218 in number that is (17.0%) of the study population  TB  Participants Younger than 15 years were 137 in number which is (11.9%) of the study while those 15–24 years were 120 in number that is (10.8%) of the study population | Health workers  providers and peer volunteers was staggered across the two clusters, following the introduction of a set of enhanced record-keeping tools | The study team developed a unit HIV testing service (HTS) register with several enhanced fields to facilitate documentation of partner/couple status and LTC of clients who tested HIV-positive. A referral logbook was developed to document LTC and re-linkage following completion of TB treatment among TB/HIV patients | Introducing the enhanced documentation was associated with increased recording of linkages from Time 0 (5.7%) to Time 1 (50.0%), but there was no additional increase associated with the training. This was not age specific | Individual level :  It is not clear how long follow-up was for the participants in this study.  Program level:  No Indicators of program level maintenance were not reported |
| Batya Elul et al (2017),Mozambique | cluster-randomized trial, 10 primary health facilities in the city of Maputo and In hambane Province were randomly assigned to provide the Combination intervention strategy (CIS) or the standard of care (SOC). | A combined outcome of increased linkage to HIV care within 1 month of diagnosis and retention in  care at 12 months across the 3 study groups. | 265 (13%) young people within the age of 18-24 years were involved in the study | Program delivered by Research assistants ,clinicians and facility  receptionist | The CIS included point-of-care CD4 testing at the time of diagnosis, accelerated ART initiation, and short message service (SMS)  patients in the CIS+ cohort received the CIS interventions plus a series of non-cash financial incentives (FIs) in the form of prepaid cellular air-time cards to offset structural barriers associated with the direct and indirect costs of coming to the health facility to receive HIV care  Air-time cards rather than cash were selected as the incentive based on discussion with the Ministry of Health. Each card was valued at approximately US$5 and was provided conditionally upon the following achievements: linkage to care within 1 month of diagnosis, retention in care 6 months after diagnosis, and retention in care 12 months after diagnosis, for a total of approximately US$15. | the greatest intervention effects were observed among young adults age 18–24 years (RRCIS vs SOC = 2.39, 95% CI 1.51–3.80, p-value for interaction between age and treatment arm = 0.07), the intervention effect was greatest in subpopulations documented to have particularly poor outcomes across the HIV care continuum, including young adults | Individual level :  Participants were followed –up for 12 months.  Program level:  No Indicators of program level maintenance were not reported |
| Hewett et al (2016), Zambia | RCT  The study was initiated at at seven health service sites  There were two types of study sites: entry point sites,  where clients were recruited into the study, and referral  sites, to which clients were referred for additional services. Each district had at least one of the three entry point services: HIV Testing and Counseling, Family Planning, and voluntary medical male circumcision,  Referral service sites included SFH-operated integrated service centers, public hospitals/clinics, and partner NGO-run service centers; all referral sites were mapped and located within walking distance of the entry point locations.. | contribute to the existing evidence base for best practices in SRH and HIV service linkage and integration, as well as to determine whether two interventions designed to enhance services provision, increase referrals to add-on services, and improve client follow-up, would increase the likelihood that clients would access additional services.  The outcomes were measured for enrolled clients, as well as their spouses/partners, if applicable. Uptake was defined as clients reporting that they or their spouse/partner utilized a referred service within the six-week or six-month period after enrollment. | 47.8% of the total study population 3963 were within the age 18-24 years | trained enumerator | The 3 arms of the study included  1)Standard of care   - Standard client assessment and counseling - Ad-hoc referrals to add-on services - No transition or linkage between services - No follow-up of clients   2)Enhanced referrals with client follow-up   - Standardized assessment of need. - Dedicated counseling time - Informational materials for add-on services - Motivational interviewing techniques - Standardized referral - Follow-up of clients   3)Enhanced referrals with client follow-up and escort  This has all that is in arm two with the addition of escort to add-on services | . Integrated services were found to be more efficiently provided than vertical service provision; the cost-effectiveness for HIV/AIDS and cervical cancer was high in the enhanced service models.  Study results provide evidence for increasing the linkages and integration of a selection of HIV and sexual and reproductive health services. The study provided cost-effective service delivery models that enhanced the likelihood of clients accessing some additional needed health services.  The embedded economic evaluation found the intervention to be highly cost effective for HTC (study arm 3 only), VMMC, HIV care and treatment and for cervical cancer screening. The study’s impact and cost-effectiveness results suggest that the enhanced service models evaluated are worthy of strong consideration when adding or integrating health services across platforms.  the cost-effectiveness for HIV/AIDS and cervical cancer was high in the enhanced service model | Individual level :  Participants were followed up at six weeks, and six months  Program level:  No Indicators of program level maintenance were not reported  clients were tracked at six weeks and six months post-enrollment for an interview that included questions about service uptake at study and nonstudy health facilities |
| Niklaus Daniel Labhardt et al (2014),south Africa Lesotho | The study was an open-label, two-armed cluster-randomizedtrial conducted in two rural catchment areas.  This trial compares HB-HTC to mobile clinic HTC (MC-HTC) | All outcomes were assessed at the individual level   1. the number of persons taking up HTC. 2. the proportion ofparticipants newly tested HIV-positive among participants whotook up HTC. 3. linkage to carewithin 28 d after a positive HIV test among participants whotested HIV-positive during the campaign. 4. chronic care within 28 d.The first secondary outcome was the age group distribution ofpersons accessing the campaigns (,12, 12–24, and$25 y). Thesecond secondary outcome was the proportion of first-time testersamong people who took up HTC. ‘‘First-time tester’’ was definedas an individual reporting never having had an HIV test beforeand taking up HTC during the campaigns | 504(43.1%) 12-24 years in the HB-HTC group  563(40.5%) in the MC-HTC group | one lay counselor and one nurse, Professional counsellor and health workers at the 12 study health centers. | Home-based HTC (HB-HTC) is a popular community-based approach to reach personswho do not test at health facilities. InterventionThe MC-HTC group provided services through ‘‘pitso’’—themore routine HTC approach in Lesotho. ‘‘Pitso’’ is a communitygathering, usually held at the chief’s place.  the HB-HTC groupthere was no community gathering or health talk.  the two sub-team started from thechief’s place and provided all the services at people’s homes, goingdoor-to-door towards the periphery of the village. Services wereusually provided to all persons living in the same household at thesame time. | OverallHTC uptake was higher in the HB-HTC group than in the MC-HTC group (92.5% versus 86.7%; adjusted odds ratio [aOR]:2.06; 95% CI: 1.18–3.60;p= 0. 011). Among adolescents and adults$12 y, HTC uptake did not differ significantly betweenthe two groups; however, in children,12 y, HTC uptake was higher in the HB-HTC arm (87.5% versus 58.7%; aOR: 4.91; 95%CI: 2.41–10.0;p,0.001). Ten (25.6%) and 19 (25.3%) individuals in the HB-HTC andin the MC-HTC arms, respectively, linked to HIV care within 1 mo after testing positive.  HB-HTC appears to be a promising approach toimprove coverage of hard-to-reach populations, such as children,first-time testers, and men—however, only if combined witheffective interventions improving linkage to care | Individual level :  Participants were followed up at 28 days after testing positive to confirm that client has been linked to care.  Program level:  No Indicators of program level maintenance were not reported |
| Joseph.K.B.Matovu et al (2020),Uganda | Cross sectional study | Increase feasiblility and acceptibility ,and achieve high linkage to HIV care among newly diagnosed HIV-positive individuals | 168 young people aged 15-24 years which is 56% of the total study population | trained“peer-leaders, | Distribution of HIV-Self testing kits to eligible social network members. Incentive of $4 was given to respondents for travel expenses. The intervention was evaluated against the feasibility benchmark of 70% of peer-leaders distributing up to 70% of the kits that they received; and the acceptability benchmark of >80% of the respondents self-testing for HIV. | Of the 57.1% (n = 12) first-time HIV-positives, 100% sought confirmatory HIV testing and nine of the ten (90%) respondents who were confirmed as HIV-positive were linked to HIV care within 1 week of HIV diagnosis | Individual level :  Participants were followed up at four weeks.  Program level:  No Indicators of program level maintenance were not reported |
| Reshma Naik et al (2015), South Africa | It was part of a largercluster randomized controlled trial of door-to-door HB-HCTcalled ‘‘Good Start,’’ | highlight barriers to linkage following an increasingly popular model of HIV testing. practical interventions and health education strategies that could be used to improve linkage to care | All HIV positive HB-HCT clients 15-24 years which is 22.4% of the study population | trained lay counsellors, Study nurse | HIV-positive clients identified through home-based HIV counselling and testing (HBHCT) were followed up to assess linkage to care, defined as obtaining a CD4 count.  during periodic home visits or phone calls, counsellors used a paper-based monitor-ing tool to record self-reported follow-up information and(2) the study nurse tracked referral letters and followed upon self-reported clinic attendance by checking and obtaining information from official registers at the health facilities. Extensive efforts were put into follow-up including at least three attempted contacts for each client, frequent visits to clinics and thorough cross-checking of facility records. | Factors predictive of decreased linkage included the following: younger age 15 to 24 years (aHR 0.50; 95% CI: 0.28 to 0.91); living with two or more adults (aHR 0.52;95% CI: 0.35 to 0.77); | Individual level :  Participants were followed up at three times within three months till they were linked to care.  Program level:  No Indicators of program level maintenance were not reported |
| Lucy Anne Parker et al (2015), Swaziland | Cross sectional study | To evaluate the feasibility (population reached, costs) and effectiveness (positivity rates, linkage to care) of two strategies of community-based HIV testing and counselling (HTC) in rural Swaziland. | Under the MHTC there were 245 adolescents 10-19 years old that is 12% of the total population. While under the HBHTC there were 1924 adolescents 10-19 years old that is 27.4% (7026) of the total population | Health workers at the ministry of health and MSF led the mobile testing | The first strategy was mobile HTC (MHTC), introduced on an ongoing basis from September 2012. MSF testing teams visited community sites identified by community leaders, attended mobile ‘outreach’ clinics and set up testing sites at major events. The second strategy was homebased HTC (HBHTC), implemented on a campaign basis in August 2013. The campaign took place in three remote communities that were sensitised ahead of time via radio announcements. During the campaign, the testers moved through the community by foot visiting the households door to door. | Although the number of those HIV positive was higher in the MHTC the number linked to care was fewer than the HBHTC group within 6 months (60(35%) Vs75(33%)), This was not stratified to the ages and though a higher number of adolescents were tested through the HBHTC it was not stated if they were linked to care. MHTC, was a more expensive strategy.  We found that HBHTC cost less than MHTC and was more effective at reaching first-time testers and people who had not tested in the past 12 months. F | Individual level :  Participants who tested HIV positive were followed up for 6 months till they were linked to care.  Program level:  No Indicators of program level maintenance were not reported |
| K du Preez et al(2020), South Africa | Prospective corhort study | Impact of a hospital-based referral service (intervention) to reduce initial loss to follow-up among children with tuberculosis (TB) and ensure the completeness of routine TB surveillance data. | Children <13 years  272 children with TB (102 [38%] culture-confirmed) were discharged to continue TB care at a community-based PHC facility (n = 244) or at the TBH outpatient department (n = 28). TB education and counselling were completed with parents/caregivers of 230 (85%) children, and referral documentation was completed for 220 (81%) children. | A hospital-based TB referral service, staffed by a dedicated full-time nursing officer and a lay healthcare worker, was established in the paediatric wards and outpatient clinics at TBH in 2012. | A dedicated TB referral service was established in the paediatric wards at Tygerberg Hospital, Cape Town, in 2012. Allocated personnel provided TB education and counselling, TB referral support and weekly telephonic follow-up after hospital discharge. All children identified with TB were matched to electronic TB treatment registers (ETR.Net/EDRWeb).  In-hospital support for children routinely diagnosed with TB by TBH clinical staff included TB education and counselling of parents/caregivers (by telephone if not possible in person), and supporting completion of routine TB referral stationary. During study implementation, paediatric hospital personnel received ongoing training and feedback regarding appropriate TB referral procedures. All intervention activities were implemented as part of an integrated package of TB care for children at TBH. Following discharge, intervention support included weekly follow-up by telephone with TB staff at the receiving PHC to confirm whether the child had accessed care, and with parents/caregivers if necessary. T | Successful referral with linkage to care was confirmed in 267/272 (98%) and successful reporting in 227/272 (84%) children.  A simple hospital-based TB referral service can reduce initial loss to follow-up and improve recording and reporting of childhood TB in settings with decentralised TB services. | Individual level :  Participants are follow-up via telephone weekly.  Program level:  No Indicators of program level maintenance were not reported |
|  |  |  |  |  |  |  |  |

| Home based counselling and testing | Facility counselling and testing | Home-based visits enhanced counseling | Follow-up phone calls and home visits enhanced counseling | Referrals to counselling,testing and care | Mobile clinic | Incentive reimbursement of transportation | escort | message service (SMS) | community health campaigns | Social network | HIVST |
| --- | --- | --- | --- | --- | --- | --- | --- | --- | --- | --- | --- |
| Ahmed,brown | Ahmed | chang, conan, LAB+Paarker | Boeke 2,bajaria, ayieko, Nalik(all) | Chang, bajaria,choko,CQ(referral log book and re-linkage to care),hewett,peeze | Lab  Parker | Ayieko,elul,matov | hewett | ELUL | brown | Brown | Choko, matov |
|  |  |  |  |  |  |  |  |  |  |  |  |
